# Supplementary material for: Structural Modelling of KCNQ1 and KCNH2 Double Mutant Proteins, Identified in Two Severe Long QT Syndrome Cases, Reveals New Insights into Cardiac Channelopathies
Source: Int J Mol Sci. 2021 Nov 28;22(23):12861. doi: 10.3390/ijms222312861 (PMC8657475; doi:10.3390/ijms222312861)
Supplement: Supplementary file 1 [file ijms-22-12861-s001.zip › SI-figureS1.pdf]

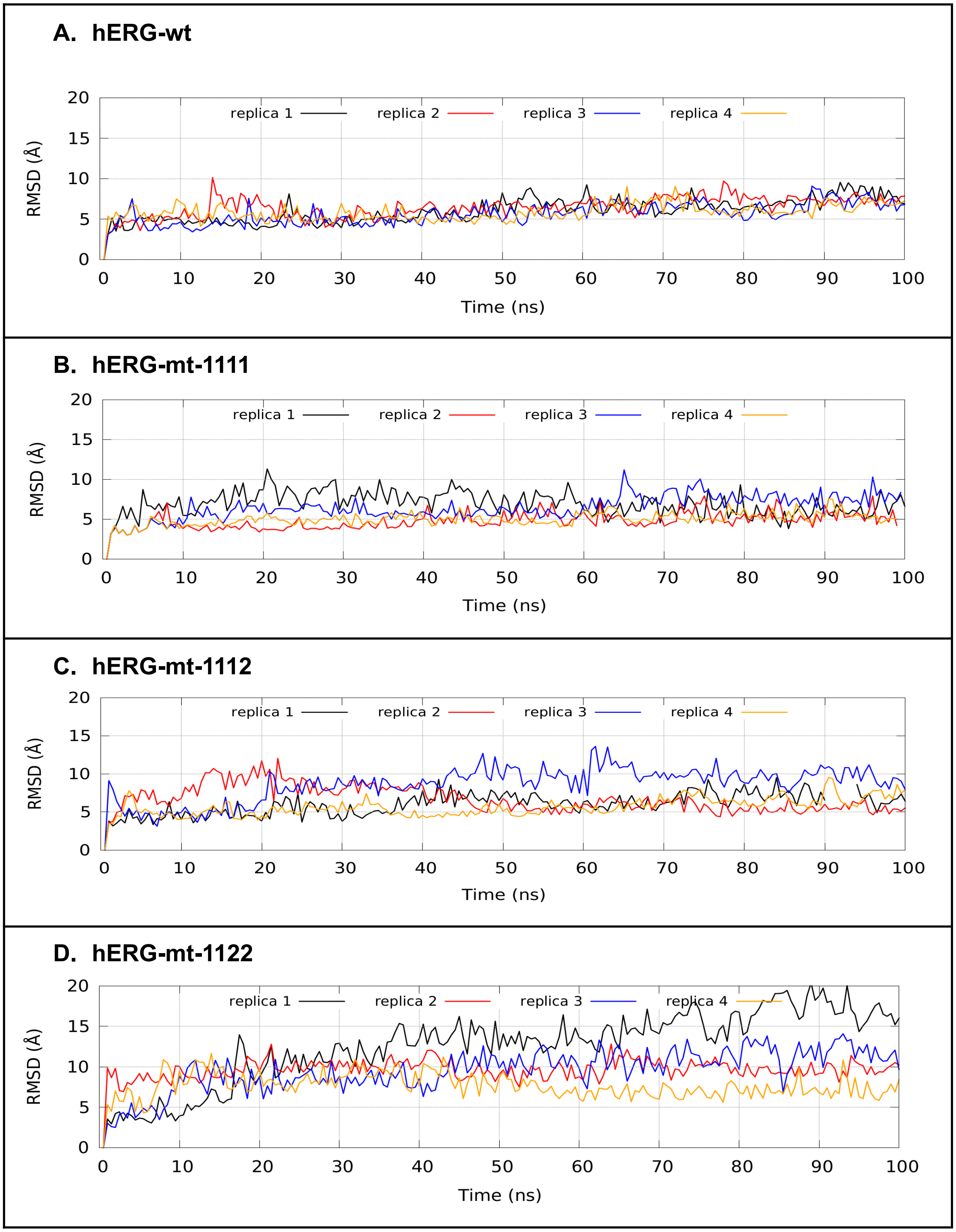
A. hERG-wt
replica 1
replica 2
replica 3
replica 4
RMSD (Å)
Time (ns)
B. hERG-mt-1111
replica 1
replica 2
replica 3
replica 4
RMSD (Å)
Time (ns)
C. hERG-mt-1112
replica 1
replica 2
replica 3
replica 4
RMSD (Å)
Time (ns)
D. hERG-mt-1122
replica 1
replica 2
replica 3
replica 4
RMSD (Å)
Time (ns)

E. hERG-mt-1212

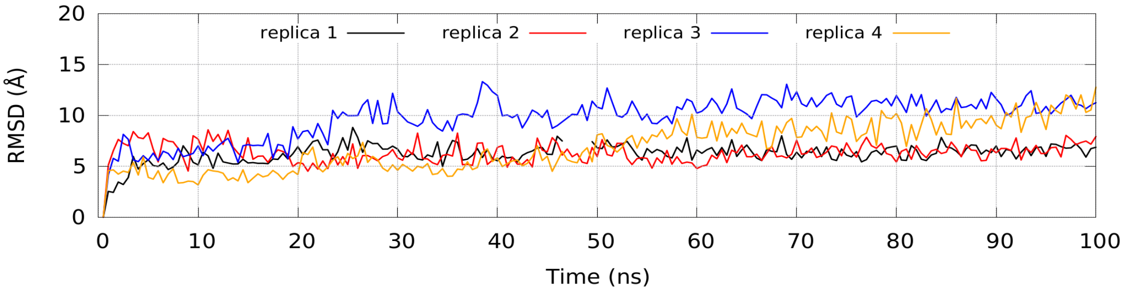

F. hERG-mt-1222

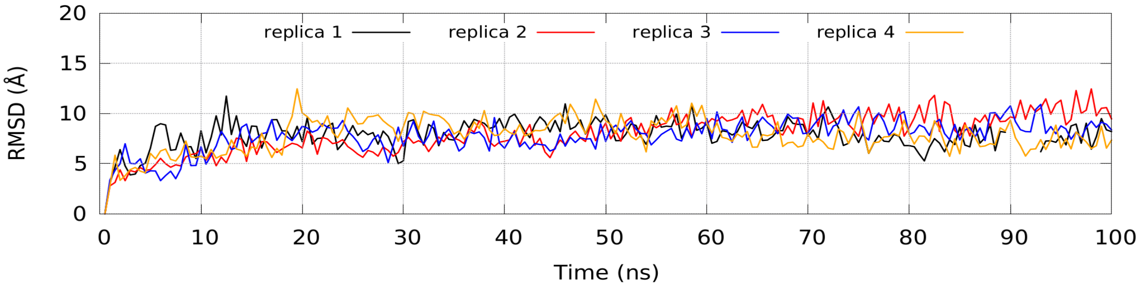

G. hERG-mt-2222

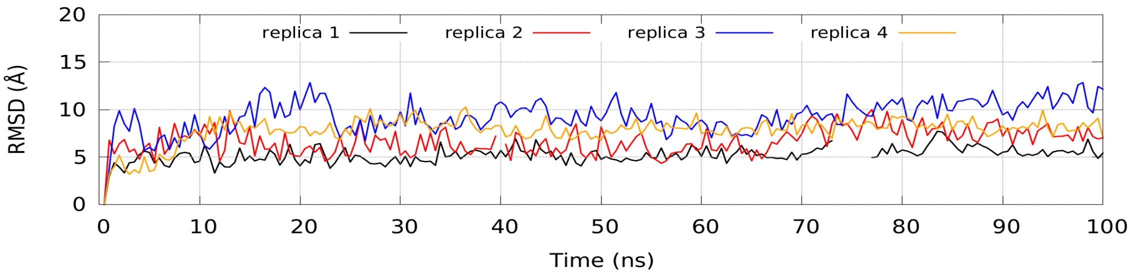

Figure S1: hERG Channel. Cα RMSD for each replica.
