# Supplementary figures and images for: Structural Modelling of KCNQ1 and KCNH2 Double Mutant Proteins, Identified in Two Severe Long QT Syndrome Cases, Reveals New Insights into Cardiac Channelopathies
Source: Int J Mol Sci. 2021 Nov 28;22(23):12861. doi: 10.3390/ijms222312861 (PMC8657475; doi:10.3390/ijms222312861)

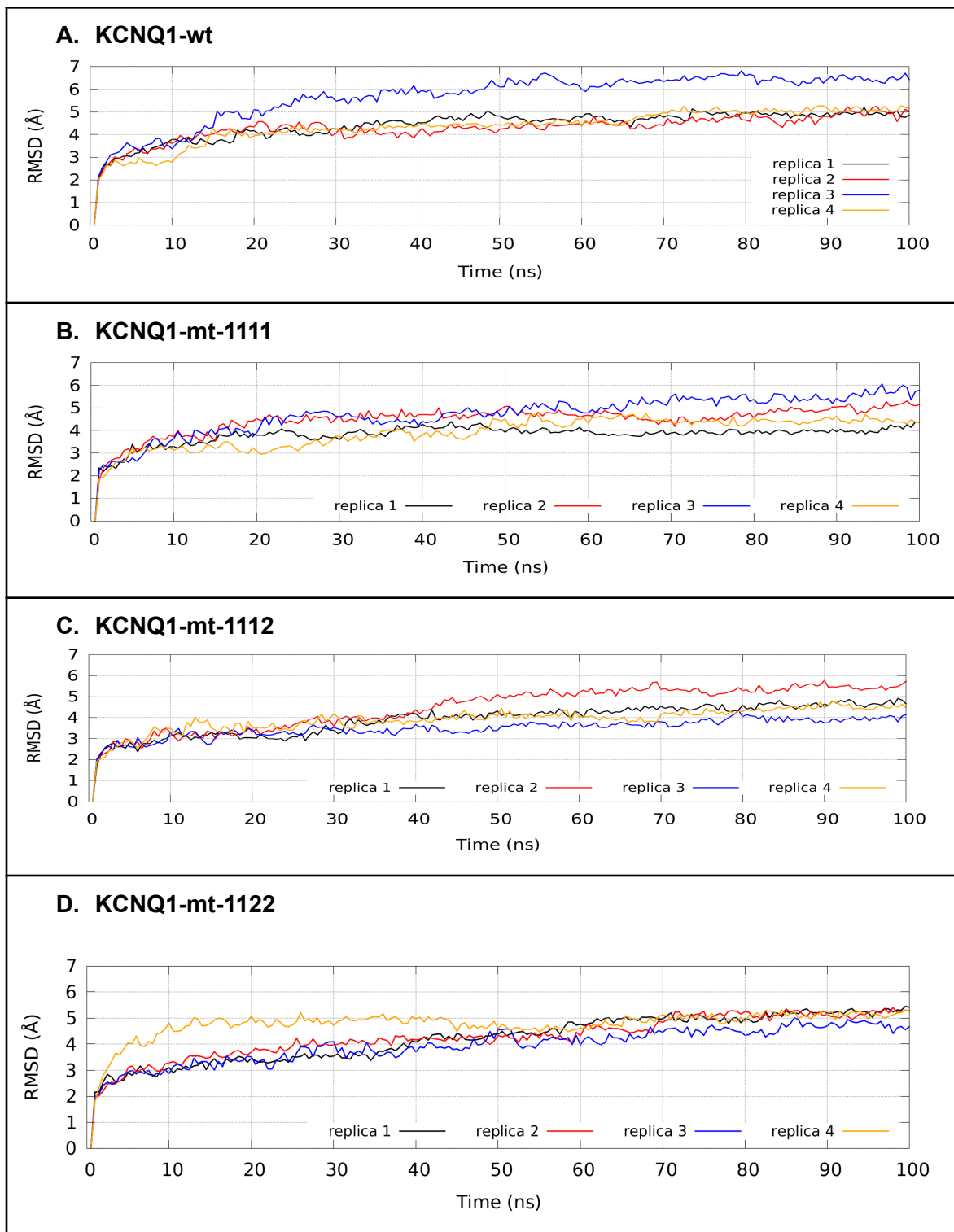

### E. KCNQ1-mt-1212

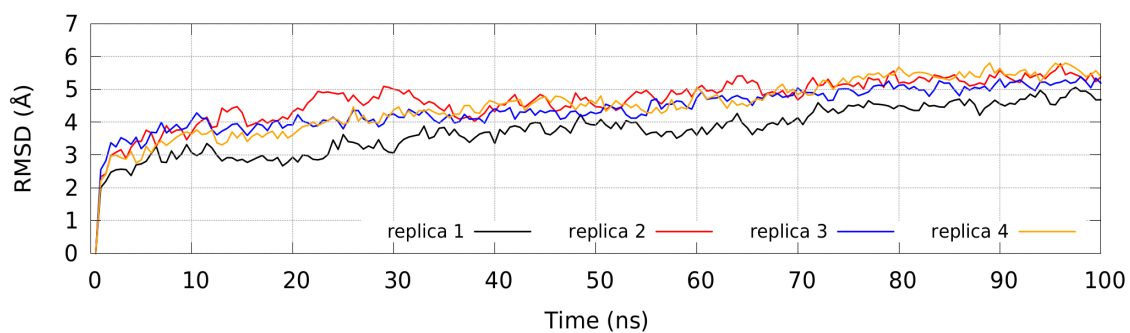

### F. KCNQ1-mt-1222

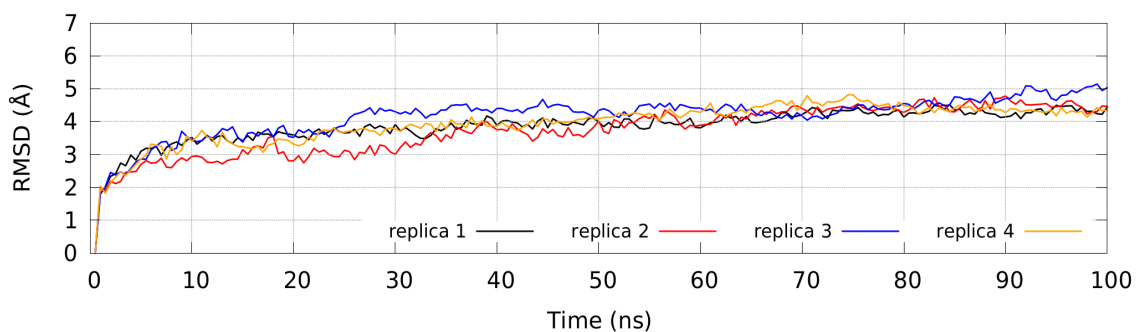

### G. KCNQ1-mt-2222

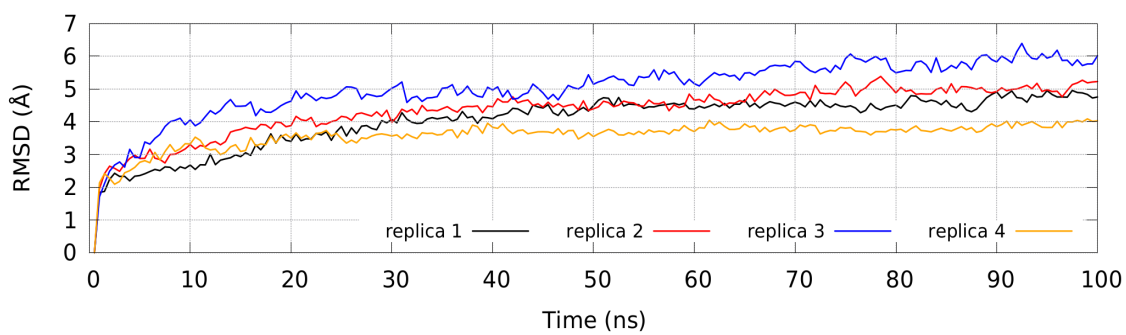

**Figure S2:** KCNQ1 channel. C $\alpha$  RMSD for each replica.

Supplement: Supplementary file 1 [file ijms-22-12861-s001.zip › SI-figureS2.pdf]

## Channel pore radius and volume

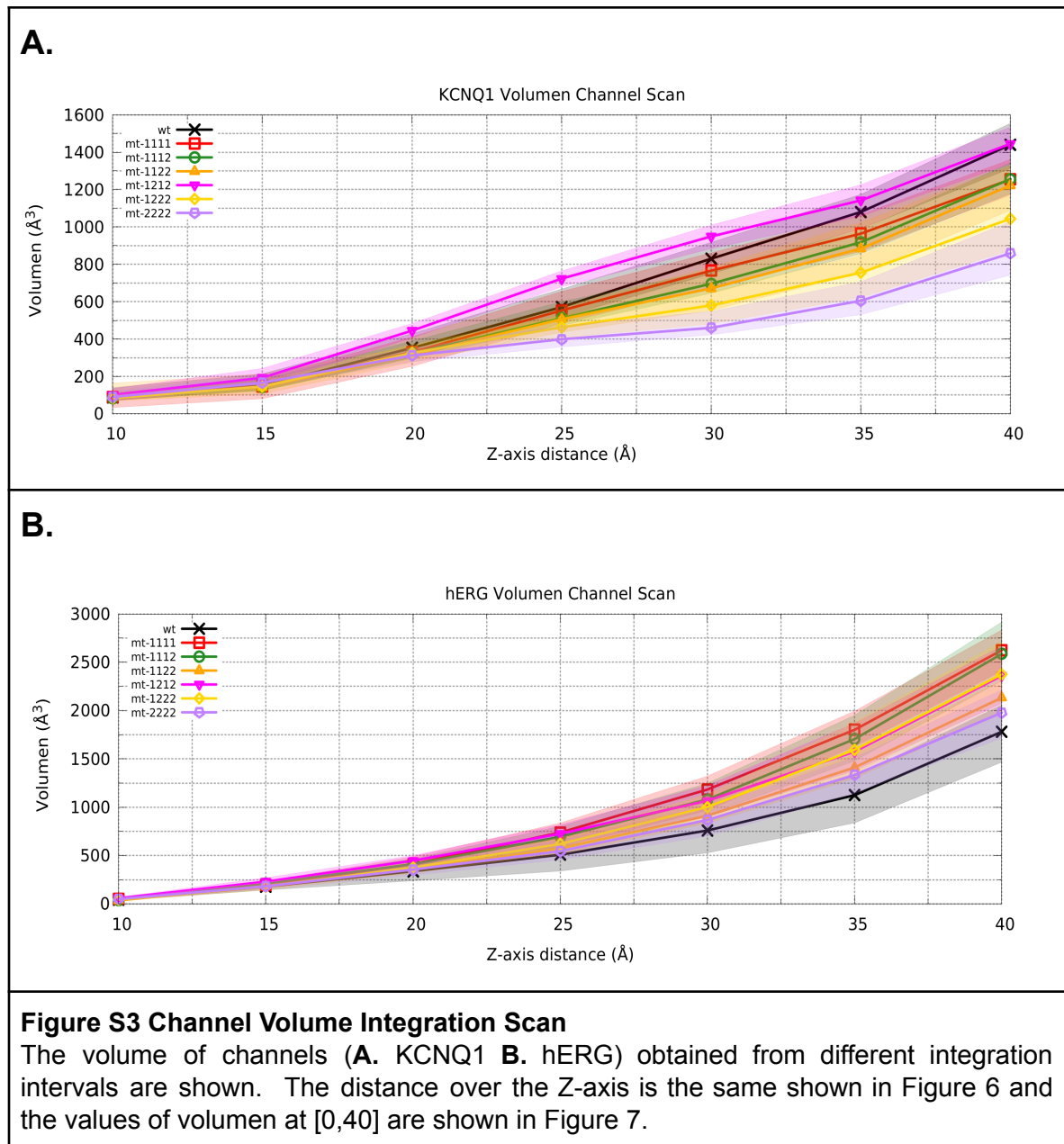

Supplement: Supplementary file 1 [file ijms-22-12861-s001.zip › SI-figureS3.pdf]
